# Medication adherence in patients with schizophrenia: a qualitative study of the patient process in motivational interviewing

**DOI:** 10.1186/s12888-018-1724-9

**Published:** 2018-05-18

**Authors:** Jos Dobber, Corine Latour, Lieuwe de Haan, Wilma Scholte op Reimer, Ron Peters, Emile Barkhof, Berno van Meijel

**Affiliations:** 1 ACHIEVE Centre of Applied Research, Faculty of Health, Amsterdam University of Applied Sciences, Tafelbergweg 51, 1105 BD Amsterdam, The Netherlands; 2 Department of Psychiatry, Academic Medical Center, University of Amsterdam, Meibergdreef 9, 1105 AZ Amsterdam, The Netherlands; 3 Department of Cardiology, Academic Medical Center, University of Amsterdam, Meibergdreef 9, 1105 AZ Amsterdam, The Netherlands; 4 GGZ Rivierduinen, Sandifortdreef 19, 2333 ZZ Leiden, The Netherlands; 5 Inholland University of Applied Sciences, De Boelelaan 1109, 1081 HV Amsterdam, The Netherlands; 6 Department of Psychiatry, VU University Medical Center, De Boelelaan 1117, 1081 HV Amsterdam, The Netherlands; 7 Parnassia Psychiatric Institute, Monterseweg 93, 2553 RJ The Hague, The Netherlands

**Keywords:** Motivational interviewing, Schizophrenia, Medication-adherence, Patient process

## Abstract

**Background:**

Motivational interviewing (MI) may be an effective intervention to improve medication adherence in patients with schizophrenia. However, for this patient group, mixed results have been found in randomized controlled trials. Furthermore, the process of becoming (more) motivated for long-term medication adherence in patients with schizophrenia is largely unexplored.

**Method:**

We performed a qualitative multiple case study of MI-sessions to analyse the interaction process affecting motivation in patients with schizophrenia. Fourteen cases of patients with schizophrenia, who recently experienced a psychotic relapse after medication-nonadherence, were studied, comprising 66 audio-recorded MI-sessions. In the MI-sessions, the patients expressed their cognitions on medication. We used these cognitions to detect the different courses (or patterns) of the patients’ ambivalence during the MI-intervention. We distinguished successful and unsuccessful cases, and used the cross-case-analysis to identify success factors to reach positive effects of MI.

**Results:**

Based on the expressed cognitions on medication, we found four different patterns of the patient process. We also found three success factors for the intervention, which were a trusting relationship between patient and therapist, the therapist’s ability to adapt his MI-strategy to the patient’s process, and relating patient values to long-term medication adherence.

**Conclusions:**

The success of an MI-intervention for medication adherence in patients with schizophrenia can be explained by well-defined success factors. Adherence may improve if therapists consider these factors during MI-sessions.

## Background

About 75% of patients with schizophrenia discontinue their antipsychotic drug treatment within 18 months [1]. Antipsychotic drug treatment reduces the risk of relapse (RR = .35), and the risk of readmission (RR = .38) [2]. It also increases the risks of a movement disorder (RR = 1.55), sedation (RR = 1.50), and weight gain (RR = 2.07) [2]. In a systematic review, Higashi et al. [3] found that lack of illness insight, beliefs about the effectiveness of medication, substance abuse, and the quality of the therapeutic relationship were important influencing factors. Enhancing patient motivation, by taking into account these factors, may be key to encouraging medication adherence.

Motivational Interviewing (MI) is an effective intervention to enhance motivation for behavioural change [4–8]. MI has been investigated as an intervention for medication adherence problems in patients with psychotic disorders. Although the results are mixed, MI shows promising results in several studies [9–12].

MI-interventions comprise four overlapping processes: engaging (establishing a trusting relationship), focusing (determining the target behaviour for change), evoking (eliciting patients’ own good motives in favour of change: “change talk”), and planning (helping to move on to actual change) [13]. In MI-theory three critical components of motivation comprise (1) willingness/importance, (2) ability/confidence and (3) readiness to change [14]. Patients are often ambivalent, expressing conflicting motivations towards change. Supporting the patient to solve this ambivalence is an important task of the MI-therapist [13]. When applying MI, therapists intentionally influence these components to elicit intrinsic motivation and to enable behavioural change. Hereto the therapist communicates in an empathic style and with “MI-Spirit” (the core values of MI: partnership, acceptance, evocation and compassion) [13]. The “language of change” plays a major role in MI. MI-therapists elicit patient change talk in which the patient hears her/himself argue for change. Meanwhile therapists try to avoid “patient sustain talk” (in favour of status quo). To be effective, the therapist tunes the MI-strategy to the patient’s process of becoming more motivated. Specific knowledge of the nature of this process in patients with schizophrenia may help practitioners to improve their tuning of the motivational process in the MI-sessions and enhance the effects of MI for sustained medication adherence. Therefore, the aim of this current study is to explore the patient process of becoming (more) motivated in a group of patients with schizophrenia, who recently experienced a psychotic relapse after medication-nonadherence. We address the following questions: (1) Can different patterns of the patient process be distinguished in patients with schizophrenia? (2) Can successful cases be distinguished from unsuccessful cases? (3) How do successful cases differ from unsuccessful cases?

## Methods

### Study design

We used a qualitative multiple case study [15] to discover and explore the patient’s motivational process during MI-sessions. This design is an inductive interpretative study of cases, to promote understanding of psychosocial processes influencing the patient process of becoming motivated for long-term medication use.

The multiple case study analysis comprised three phases: single case analysis, cross case analysis, and cross case synthesis [15]. Each case consisted of (1) audio records of at least three MI-sessions, (2) coded transcripts, (3) global ratings of the therapist style and the patient self-exploration in each session, and (4) summary scores measuring therapist MI-fidelity.

### Study population

The cases were derived from the intervention group of a Randomized Controlled Trial investigating the effect of MI on medication adherence in non-adherent patients with multi-episode schizophrenia, who had experienced a recent psychotic relapse, following nonadherence to antipsychotic treatment [9]. The 55 participants in the intervention group were offered up to nine MI-sessions to promote motivation for medication-adherence.

### Data collection and analysis

In the original trial, MI-sessions were audio-recorded if the participant consented to this. In the present study, patients were included if there were at least three sessions audiotaped, and if the patient did not experience active psychotic symptoms (as demonstrated by dominant verbal references to current hallucinations or delusions) during the MI-intervention. Five therapists were involved: a psychiatrist, a psychologist, and three community mental health nurses. Neither of the therapists had previous experience in MI. They followed a 32-h MI training by a certified MI-trainer (member of the Motivational Interviewing Network of Trainers), and participated in monthly supervision on MI-fidelity during the conduct of the trial.

All audio recordings were transcribed verbatim, and parsed into patient and therapist utterances. For coding, we used a combination of the Motivational Interviewing Sequential Code for Observing Process Exchanges (SCOPE) [16] and the Motivational Interviewing Skill Code 2.1 (MISC 2.1) [17] (Table 1). For each session, summary scores were computed to assess the quality of MI-execution [16–18]. The first author was trained in MISC-coding at the MI-coding lab of the Center for Alcohol and Addiction Studies, Brown University, USA. He subsequently trained the two coders (one master level, one bachelor level) for performing data-analysis in the present study. After a 37-h training the coders reached a Kappa of .82 on behaviour codes. For the global ratings, we considered a maximum of one point difference on the 7-point scales as an agreement, and a difference of more than one point as a disagreement. So, we dichotomised the scores to “agreement” and “disagreement” (see also Kaplan et al. [19]). After the training, the coders reached a Kappa of 1.0 on the global ratings. Transcripts were first broken down into separate encodable utterances (“parses”) by one coder. A second coder then coded the transcript in two passes. In the first pass, the coder listened uninterruptedly to the complete session, assigned the global ratings, and registered the optional MI-components (Table 1). In the second pass, each parse was coded in one of the coding categories (Table 1). Coding dilemmas were solved in weekly coder-trainer meetings. We randomly selected 10% (*n* = 7) of the sessions for recoding by the same coder to verify intra-rater agreement (Kappa behaviour codes = .77; Kappa global ratings = 1.0), and 20% of the sessions (*n* = 13), randomly selected, were double-coded for interrater agreement (Kappa behaviour codes = .71; Kappa global ratings = .84).

**Table 1:** Measures and coding instruments

Unit of measurement	Measurement	Coding instrument
Therapist	sequential coding of 20 verbal behaviours: question, reflection, advice with permission, permission seeking, affirm, emphasize control, support, advice without permission, confront, direct, opinion, raise concern, warn, facilitate, feedback, filler, self-disclosure, general information, structure, not encodable.	SCOPE (Motivational Interviewing Sequential Code for Observing Process Exchanges) [16]
	rating of 5 MI-core values and other relational ingredients on a 7-point global rating scale: acceptance, empathy, collaboration, evocation, autonomy.	MISC 2.1 (Motivational Interviewing Skill Code) [17]
	computing 5 summary scores: • ratio of reflections to questions, • percent open questions, • percent complex reflections, • percent MI-consistent techniques, • mean global ratings.	MITI 3.1.1 (Motivational Interviewing Treatment Integrity) [18] SCOPE SCOPE SCOPE SCOPE MISC 2.1
	registration of optional MI-components: decisional balance, importance ruler, confidence ruler, typical day/week, looking back, looking forward, exploring goals and values, querying extremes, developing change plan.	Registration: applied / not applied
Patient	sequential coding of 10 patient verbal behaviours: commitment, desire, ability, reasons, need, taking steps, other, ask, follow neutral, not encodable.	SCOPE
	rating of the level of patient self-exploration on a 7-point global rating scale.	MISC 2.1
	percent patient change talk.	SCOPE

During the multiple case analysis, a detailed log was kept on the research process, the findings, and the decisions. The analyst (JD) used worksheets based on Stake [15] to structure the analysis, and composed case reports. Two other investigators (CL, BvM) independently scrutinized random subsets of these materials, to ascertain the appropriateness of the research process, and to assure the integrity of the findings, decisions, and conclusions. Also, another investigator independently double analysed two cases. In case of disagreement, the original data were checked and disagreements were resolved by discussion.

### Measurement of the motivational process

We considered shifts in the cognitions on medication during the MI-sessions as indicative for changes in the patient process of motivation for long-term medication adherence. The course of these cognitions during the MI-sessions was used to determine the pattern of the patient motivational process. We assumed that a trusting relationship between patient and therapist supports the patient to open up and talk freely about his/her experiences, goals, values, concerns and ambivalence related to medication adherence. This patient self-exploration is measured by a 7-point global rating scale [17]. We regarded a score of four or higher on this scale as an indication of a trusting relationship.

We deduced criteria to distinguish successful and unsuccessful cases from the aim of the MI-intervention in the original study, i.e. to enhance motivation for long-term medication adherence [9]. In this intervention, the therapist should support the patient to find and explore his/her reasons and motives for medication use, and help to relate medication adherence to the patient’s values and goals. If at baseline the patient felt ambivalent about his/her long-term medication, the patient and therapist should explore this ambivalence, and, if appropriate, potential barriers. Hence, the patient may be able to solve the ambivalence or may find ways to handle perceived barriers in relation to medication adherence, based on intrinsic motivation. If the patient is not ambivalent, but takes a convinced position pro or against long-term medication use, the intervention should concentrate on either potential barriers and strengthening long-term motivation for medication adherence (in case of a motivated patient), or exploring possible goals and values in relation to medication adherence to find out if new perspectives on medication adherence can be evoked (in case of no motivation). Thus, three criteria for success applied to all cases, while other criteria depended on the baseline ambivalence and motivation (Table 2).

**Table 2:** Criteria for success

Criteria	Ambivalent at baseline	Not ambivalent at baseline	
		motivated for MA^a^	no motivation for MA^a^
During the MI-sessions the patient has seriously considered what his/her motives are (not) to adhere to long-term medication.	X	X	X
Existing ambivalence and/or potential barriers are explored.	X	X^b^	
Values and goals are explicitly discussed in relation to medication adherence.	X	X	X
The patient solved the ambivalence and/or has an action plan for perceived barriers.	X		
Long-term motivation was strengthened.		X	
The decision (not) to adhere is based on intrinsic motivation: the patient articulates the intention (not) to adhere to long-term medication, based on motives that are valid to the patient.	X	X	X

^a^MA = Medication adherence

^b^Exploration of potential barriers

Finally, we compared the determined patient motivational processes with the outcomes on the medication adherence item of the Life Chart Score [20] (LCS, range 1–5, higher scores indicating higher levels of adherence) in the original RCT at 6-month follow-up [9].

## Results

The inclusion criteria led to a sample consisting of 66 audiotaped sessions of 14 of the 55 participants of the original trial. The participants’ background characteristics are listed in Table 3. Based on MI-theory, we distinguished eight possible patterns of the patient process (Table 4).

**Table 3:** Background characteristics

	Number (%) *n* = 14
Gender: male	10 (71%)
Age: mean (range)	35.5 (23–48)
21–30	4 (28.5%)
31–40	6 (43%)
41–50	4 (28.5%)
Ethnicity	
Dutch	6 (43%)
Surinamese	4 (28.5%)
African	3 (21.5%)
Asian	1 (7%)
Native language is Dutch	
Yes	9
No	5
Highest education	
primary education or less	2 (14%)
secondary education	10 (71%)
tertiary/further education	2 (14%)
Duration of illness: mean in years (range)	6,9 (1–23)
Number of prior psychiatric admissions: mean (range)	3,4 (0–8)
Diagnosis (subtype schizophrenia, DSM-IV)	
disorganized type	2
paranoid type	6
residual type	1
undifferentiated type	1
schizoaffective disorder	4

**Table 4:** Patterns of the patient process

Baseline	Development of patient process during MI-sessions			Observed cases in this pattern
Not-ambivalent	Remained not-ambivalent		Motivation for medication adherence	cases 9, 10, 11, 12
			No motivation for medication adherence	cases 3 and 7
	Became ambivalent	Ambivalence, solved	Motivation for medication adherence	no cases
			No motivation for medication adherence	no cases
		Ambivalence, not solved		no cases
Ambivalent		Ambivalence, solved	Motivation for medication adherence	cases 5, 13, 14
			No motivation for medication adherence	no cases
		Ambivalence, not solved		cases 1, 4, 6, 8

Based on 13 cases. The pattern in case 2 remained unclear

All patients expressed cognitions on medication use. Overall, 213 cognitions were classified in four categories: (1) cognitions supporting motivation for long-term medication use (*n* = 90); (2) cognitions containing reasons to stop (*n* = 58); (3) cognitions reflecting doubt or ambivalence (*n* = 19); and (4) other cognitions (*n* = 46). Based on the course of the expressed cognitions during the MI-sessions, we detected four of eight theorized patterns of the patient process of becoming motivated (Table 4). In one case, we were not able to detect a pattern because the patient avoided serious conversations regarding medication adherence. Four cases followed the pattern ‘Ambivalent – not solved’. In the nine other cases, the patient was not ambivalent (six cases) or resolved his/her ambivalence during the MI-sessions (three cases).

Based on the criteria, we considered four cases to have run through ‘a successful patient process’ (Table 5). Hereafter we first discuss the similarities and differences between the four patient processes that we observed, and next we discuss the characteristics of the successful and unsuccessful cases.

**Table 5:** Successful and unsuccessful cases

Criteria	Serious and explicit consideration of motives	Exploration of ambivalence and/or potential barriers	Explicit discussion of values and goals in relation to MA^a^	Ambivalence was solved and/or action plan was made	Strengthening of long-term motivation	Decision based on intrinsic motivation
Cases with ambivalence at baseline						
1	+	–	–	–		+
4	+	–	–	–		–
5	+	+	+	+		+
6	+	–	–	–		–
8	+	–	–	–		–
13	+	–	–	+		–
14	+	+	+	+		+
Cases without ambivalent at baseline, motivated for MA						
9	+	-^b^	–		–	–
10	+	+^b^	+		+	+
11	–	-^b^	–		–	–
12	+	-^b^	–		–	–
Cases without ambivalence at baseline, no motivation for MA						
3	+		+			+
7	–		–			–
Case in which the client avoided a serious conversation on MA						
2	–	–	–	–	–	–

^a^MA = Medication adherence

^b^Exploration of potential barriers

+ means: this criterion was met during the MI-sessions

- means: did not meet this criterion during the MI-sessions

### Pattern 1: Not ambivalent, motivated for medication adherence

In this pattern, the four patients (cases 9, 10, 11 and 12) have in common that, from the start of the intervention, they expressed cognitions that support motivation for long-term medication use (Table 6a). So, at first glance, they don’t seem to need the MI-intervention. However, the task of the MI-therapist is also to maintain and strengthen motivation and explore potential barriers. This only happened in case 10 (successful case), where the therapist guided the patient to explain how he stays in control, and how medication helps him “to have a better life”. The patient stressed the value of this argument for medication adherence: “even if I’ll have to use medication four more years”, thus strengthening his long-term motivation.

**Table 6:** Examples of courses of cognitions on medication through the sessions^a^

*a: Pattern 1 Not ambivalent, motivated for medication adherence (case 12)*	
Session 1 • I fear the way people, like colleagues or a potential partner, will look at me if they know I’m being treated. • I’m not going to quit medication. Session 2 • Medications prevent me from experiencing a relapse in psychosis. • For the time being I need medication.	Session 3 • Provisionally, I’ll stay on medication, I might never quit. • There are so many people taking medication. Session 4 • No cognitions on medication expressed in this session.
*b: Pattern 2 Not ambivalent, no motivation for medication adherence (case 7)*	
Session 1 • The medication causes me a lot of trouble, makes me tired and gives me too much saliva. • I don’t think those medications are important for me. Session 2 • This medication is bad, I don’t need it, I quit using it. • I’m fine if I don’t use medication.	Session 3 • Sometimes, medication is important. • When I live at home, I won’t use medication. Session 4 • I want to quit medication, I’m fine.
*c: Pattern 3 Ambivalence solved, motivated for medication adherence (case 14)*	
Session 1 • It makes sense to take medication and I need it, but the dose should not be too high. • Medications have effect, but they also cause side effects. When are the gains bigger than the harm? Session 2 (no audio track available)	Session 3 • Medication makes me feel less myself. • Medication helps me to experience positive periods of time. Session 4 • Medications should be used wisely, I should not experiment with it. • I need to use this medication dose because the impact of psychosis on my life is so big, so I need to prevent that from happening.
*d: Pattern 4 Ambivalent, not solved (case 4)*	
Session 1 • When things are going better, I stop taking my pills. • When I’m feeling fine, this is not just caused by the pills, but also because I’m taking good care of myself. Session 2 • No cognitions on medication expressed in this session. Session 3 • I’m not sure if the medications have an effect. • I don’t want to be a guinea pig by unceasing changing of my medication, not knowing their effects. Session 4 • It would be much easier to accept medication if it didn’t cause side effects.	Session 5 • No cognitions on medication expressed in this session. Session 6 • In the long-term, medication is addictive. • I’m certain that quitting medication won’t make me relapse. Session 7 • No cognitions on medication expressed in this session. Session 8 • I fear medication-addiction because of long-term use. • If I were in control, it would be fine to use the medication for one more year. Session 9 • I’m not sure whether the pro’s weight out the cons.

^a^Cognitions are explicitly or implicitly expressed by the patient. Sessions may have contained more cognitions, for reasons of space limitation we used maximal two cognitions per session

### Pattern 2: Not ambivalent, no motivation for medication adherence

This patient’s process is characterized by dominating cognitions, through all MI-sessions, on reasons to stop medication (Table 6b). In one case a language barrier hindered the execution of MI, and the therapist and patient failed to engage with each other (case 7). In the other case (case 3, successful case) therapist and patient explored both the patient’s motives to stop the medication as well as alternative perspectives on the benefits of medication. However, this conversation did not evoke new cognitions on medication. As a result, the patient held on to his decision to stop the medication as soon as possible.

### Pattern 3: Ambivalence solved, motivated for medication adherence

In three cases (5, 13 and 14) the patients’ cognitions switched during the MI-sessions from doubt and ambivalence to needing long-term use of medications because of their effects (Table 6c). In the cases 5 and 14 (successful cases), this happened after exploring both sides of the ambivalence. Guided by the therapists’ open-ended questions and complex reflections, these patients discovered the relations between medication adherence, indirect benefits of medication and of relapse prevention, and important goals and values for them. This seemed to be key for the patients in solving their ambivalence. One patient saw medication as a strong protector against psychosis, but she felt that medication influenced her emotions, feeling “a little muted” and “not feeling completely myself”. However, “Keeping my job” and “Autonomy” were important values for her, as she wanted to stay in control, and so she employed a self-developed minimal dosing strategy. But sometimes the dose was too low, resulting in a relapse. Through the sessions this ambivalence shifted to “If I use an optimal dose keeping me stable, and helping me to function well in my job, I can learn to accept that I am a little muted and a little slower.” (case 5).

In case 13, during the last session, the patient also switches his cognitions from ‘doubt/ambivalence’ to ‘needing medication for its effect’. But this switch had not been preceded by an exploration of the ambivalence by the patient, nor was medication linked to the patient’s values. Hence, the base of intrinsic motivation for this patient’s decision to adherence is unclear.

### Pattern 4: Ambivalent, not solved

These four patients (cases 1, 4, 6 and 8) expressed cognitions showing doubt and ambivalence (Table 6d). In two cases (cases 4 and 8) the patient and therapist did not succeed in building a trusting relationship, and their conversations remained superficial. Both patients accepted the present need to take medication because external factors (the treating physician; being subjected to compulsory treatment) demand this. But they also set a one year limit as an acceptable period of time for medication use, with the intention to stop.

In all four cases the patient process stagnated after expressing the ambivalence towards, or barriers for, long-term medication use. The therapist and the patient kept going around in circles about the pros and the cons, and were not able to explore and solve the ambivalence.

### Successful/unsuccessful

In the first session of all cases the therapist made an effort to engage with the patient, mostly by asking the patient to review his/her illness history and his/her experiences in mental health care. A trusting relationship is the base of motivational interviewing, and therefore a prerequisite for success. In all four successful cases (3, 5, 10 and 14) and in five of the nine unsuccessful cases (1, 6, 9, 12 and 13), the MISC-rating of patient self-exploration was ≥4, indicating a trusting relationship. In the four cases (4, 7, 8 and 11) lacking such engagement, the conversation remained superficial, with limited openness shown by the patient. One patient expressed this during the last session in a closing remark: “I know what you’re thinking and what you want to say. I will not argue over that, but I have my own vision and opinion.” (case 4). In line with MI-theory, the trusting relationship was fostered if the therapist showed good listening skills, asked open-ended questions, reflected the patient’s experiences and perceptions and showed empathy, acceptance and understanding. By contrast, the relationship between patient and therapist was hindered by the therapist focusing on the actual facts in the patient’s story (ignoring the patient’s perception), and taking up the expert-role. Moreover, the emergence of a strict question-answer pattern, or the existence of a language barrier between patient and therapist, also impeded this relationship.

In successful cases the patients had the opportunity to tell their story from their perspective and without rushing. This story included ambivalence or potential barriers to long-term medication use, and the patients became aware of their ambivalences. The therapists and patients explored both sides of the ambivalence, and the patients linked medication use to their own goals and values. Hereafter the patients articulated their intention to adhere, based on their previously explored motives. In the unsuccessful cases, although articulated by the patient, ambivalences or barriers remained unexplored. In most of the unsuccessful cases the patients expressed their values, but the therapists missed opportunities to elicit change talk by linking these goals or values to medication adherence (Table 7). One patient expressed his fear of relapse and hospitalization: “I don’t ever want to go back there”, the therapist then only reflected this goal, failing to query how medication might contribute to this (case 8).

**Table 7:** Content of the MI-sessions

Elements of the sessions	Successful cases (*n* = 4)	Unsuccessful cases (*n* = 9)
	yes / no	yes / no
Trusting relationship	4 / 0	5 / 4
Open conversation on medication adherence	4 / 0	5 / 4
Ambivalence or barriers articulated by the patient	2 / 2	7 / 2
Values or goals articulated by the patient	4 / 0	8 / 1

### Medication adherence

One of the main outcomes on medication adherence after six months in the originating RCT [9] was a 5-point adherence item of the Life Chart Schedule (LCS) [20], judged by patient, physician and/or caregiver. This follow-up score was complete for six patients. The scores indicate, in accordance with their patient process, ≥4 for patients who decided to adhere to long-term medication. An exception is the ambivalent patient in case 1, with a higher score than expected (Table 8).

**Table 8:** Medication adherence at 6-month follow-up

Baseline	Patient process		Observed cases (*n* = 13)	LCS-score patient^a^	LCS-score physician^a^	LCS-score carer^a^
Not-ambivalent	Remained not-ambivalent	Motivation for medication adherence	case 9	4	–	–
			case 10	–	–	–
			case 11	5	5	5
			case 12	4	5	5
		No motivation for medication adherence	case 3	–	–	–
			case 7	–	–	–
Ambivalent	Ambivalence, solved	Motivation for medication adherence	case 5	5	–	5
			case 13	–	–	–
			case 14	–	–	–
	Ambivalence, not solved	Ambivalent on medication adherence	case 1	4	4	–
			case 4	–	–	–
			case 6	3	2	2
			case 8	–	–	–

^a^LCS = Life Chart Score-adherence item [20]. Judged by patient, caregiver and/or physician. Score 1 = prescribed medication never taken; score 2 = took less than 50%; score 3 = took more than 50%; score 4 = nearly always took the prescribed medication; score 5 = always took the prescribed medication

## Discussion

In this study, we found four patterns of the patient process of becoming (more) motivated for long-term medication adherence. We detected that the content and course of the expressed cognitions on medication may serve as a possible indicator for that process, and we identified three success factors.

The first success factor was the trusting relationship. The establishment of such a relationship promotes the depth of patient engagement. The second success factor was the therapist’s ability to adapt the MI-strategy to the patient process. Through this strategy, the therapist stimulates the mechanisms of change within the patient [21]. One of these potential mechanisms is “change talk”, as it results in the patient hearing him/herself argue for medication adherence [22]. By hearing him/herself articulating “I must take my pills, or else it will get me in trouble”, the patient strengthens his/her belief in this cognition, and fosters a self-perception [23] of being “someone who takes medication for good reasons”. In this study, we found that the lack of such a strategy in the unsuccessful cases appeared to hinder the progress of the patient process.

An explicit conversation regarding the patient’s values or goals in relation to medication adherence was the third success factor. Taking medication is often associated with being ill and not feeling well, so intrinsic motivation for long-term medication use can only be elicited if the medication serves an important goal for the patient. This means that the therapist should support the patient to reflect on his/her goals and values and on his/her willingness and ability to change (i.e. take medications for a prolonged period of time) for these goals or values. In line with MI-theory, it is the patient who has to voice this relation, not the therapist.

The combination of these success factors may constitute good MI-practice on medication adherence in this patient group. Miller and Rollnick [13, 24] stress that, in MI, the intervention comprises both the relational and technical components, and that the active ingredients must be present. But the exact effects and active ingredients may differ between target groups [22]. These active ingredients however, are not well known and based on MI-theory, which was inductively derived from the analysis of clinical practice [25, 26], and on inconsistent findings from deductive empirical research [25]. In alcohol dependency studies, change talk and intention to change were related to better outcomes [21], while therapist MI-inconsistent behaviour was related to worse outcomes [21]. In a meta-analysis on the potential technical MI-key components [27], MI-consistent skills were associated with more change talk, and MI-inconsistent skills with less change talk and more sustain talk. In a secondary analysis of two RCT’s on brief MI in college students [28], client self-exploration and therapist MI-Spirit were associated with better outcomes. In research with mixed mental health groups [29], patient engagement was found as a potential mechanism of change. So, all these studies suggest potential active ingredients that are in line with MI-theory. We found that MI-Spirit and patient engagement constituted the basis of a fruitful MI-session. Empathy, partnership and acceptance and the technical MI-strategy were essential components in the successful cases. Lane [30] and Hilton et al. [25] however, point out that the focus on theorized ingredients may be premature, and call for qualitative inductive research for a deeper understanding of the phenomenon of MI and processes within MI. In our qualitative study, we found three success factors, and two of them, “a trusting relationship” and “the therapist’s ability to adapt the MI-strategy to the patient process”, refer to MI-skills that, in line with MI-theory, are standard elements of all MI [24]. The third success factor “exploring values” is an optional MI-component, and our study suggests that this optional component may be a key component of MI in this patient group with medication adherence as the target behaviour.

Our study adds to the scarce research literature on MI to enhance medication adherence in patients with schizophrenia. Drymalski and Campbell [31] conclude in their review that, due to serious methodological concerns, there is no reliable research on MI and medication adherence in patients with schizophrenia before 2006. In the trial from which the present sample originates, Barkhof et al. [9] found no effect of MI on medication adherence, but there were indications that targeted use of MI might be beneficial for medication adherence for some subgroups. In the present study, however, we used other criteria to detect successful cases, i.e. not medication adherence per se, but a patient decision (not) to adhere, based on intrinsic motivation after explicit exploration of motives, goals, values, and solved ambivalence and potential barriers. As a consequence, one case in which the patient decided not to adhere to long-term medication use, is also a successful case (case 3).

### Strengths and limitations of this study

A strength of this study is the pragmatic character of the original RCT. After a 32-h training and with monthly supervision, the therapists started the MI-intervention. This closely parallels usual practice in non-research conditions. Hence, the results reflect the MI-practice of newly starting MI-therapists at beginning proficiency, and not of experienced MI-therapists at expert level. This is also a limitation, because it may have led to less variation in patient process patterns, and it may explain why none of the initially not-ambivalent patients became ambivalent during the MI-intervention. Another strength is the inclusion of patients with a severe course of schizophrenia who experienced a psychotic relapse due to medication nonadherence in the past year.

A limitation of this study was the size and the composition of the sample. We retrieved sufficient audio-recorded MI-sessions for 14 of the 55 patients. This led to a selection of patients from the original sample, so this study lacks an analysis of patients not-consenting to audio-recording, and of drop-out patients.

The qualitative design and the limitations in sample size and sample composition call for prudence in generalization of the findings. Many factors influence the patient process, e.g. patient factors like education level and type and severity of mental illness, therapist factors like experience, but also health care related factors such as length of time available for the MI-sessions. We studied 14 specific cases, which enabled us to gain a more profound insight in what happened in these MI-sessions. However, this does not mean these sessions represent all patient situations, so this limits the generalization of our findings. Despite these limitations, our study results offer an indication on processes that might also be important in MI-sessions with comparable patients with multi-episode schizophrenia and recent medication nonadherence in their history.

## Conclusions

First, there are different patterns of patient processes in MI on medication adherence. This suggests that motivation for medication adherence may be improved if MI-therapists adapt their MI-strategy to the process. An indicator of these processes may be found in the course of the expressed cognitions on medication.

Second, criteria based on both MI-theory and good practice of care may be useful to differentiate between successful and unsuccessful cases. Third, the findings in our study suggest that the content of a successful MI-intervention for this target behaviour comprises a trusting relationship, the articulation of ambivalence or possible barriers for sustained medication use and the exploration of this ambivalence and barriers in relation with patient values and goals. When a patient is not ambivalent, MI may support the exploration of medication adherence in relation to the patient’s values and goals, to strengthen long-term motivation, or to explore the possibility of new patient perspectives on indirect benefits of long-term medication use.

## Abbreviations

DSM-IV: Diagnostic and Statistical Manual of Mental Disorders 4th Edition
LCS: Life Chart Score
MA: Medication adherence
MI: Motivational Interviewing
MISC: Motivational Interviewing Skill Code
MITI: Motivational Interviewing Treatment Integrity
n.a.: not applicable
RCT: Randomized Controlled Trial
RR: Risk Ratio
SCOPE: Motivational Interviewing Sequential Code for Observing Process Exchanges
USA: United States of America
